# Clinical utilization of methylprednisolone in conjunction with tranexamic acid for accelerated rehabilitation in total hip arthroplasty

**DOI:** 10.1186/s13018-023-04249-8

**Published:** 2023-10-04

**Authors:** Zuqi Huang, Huazhang Dong, Changping Ye, Zhuan Zou, Weiliang Wan

**Affiliations:** Department of Traumatology and Orthopaedics, Hezhou People’s Hospital, Guangxi Zhuang Autonomous Region, Hezhou, China

**Keywords:** Methylprednisolone, Tranexamic acid, Total hip arthroplasty, Accelerated rehabilitation, Anti-inflammatory

## Abstract

**Purpose:**

This study aimed to evaluate the efficacy and safety of combined methylprednisolone (MP) and tranexamic acid (TXA) in promoting accelerated rehabilitation following total hip arthroplasty (THA). We further investigated effective strategies for rapid rehabilitation post-THA.

**Methods:**

Conducted as a randomized controlled trial involving 80 patients, the study allocated subjects into two groups. The control group received saline and TXA, whereas the experimental group was administered with an additional dose of MP. Several clinical parameters, including markers of inflammation, pain, nausea, and coagulation factors, were meticulously assessed in both groups.

**Results:**

It was observed that the group receiving the MP + TXA treatment showcased significant reductions in postoperative levels of CRP and IL-6, as well as an alleviation in pain scores. Furthermore, this group demonstrated lower incidences of postoperative nausea and fatigue, facilitating enhanced hip joint mobility. Interestingly, this group did exhibit blood glucose fluctuations within the first 24 h postoperatively. However, there was no notable difference between the groups concerning transfusion rate, postoperative hospital stay duration, and coagulation profile, and no severe complications were reported.

**Conclusion:**

The findings suggest that the combined administration of MP and TXA can appreciably enhance postoperative recovery, by reducing inflammatory markers, alleviating pain, reducing nausea and fatigue, and improving hip mobility, without leading to an increased risk of severe perioperative complications. This highlights the potential role of this combined therapy in facilitating improved postoperative patient experiences.

## Background

Total Hip Arthroplasty (THA) constitutes a highly cost-effective therapeutic approach for terminal hip pathologies, as evidenced by a wide body of literature [1, 2]. This surgical intervention dramatically ameliorates hip joint function and enhances patient quality of life. According to extant predictive models [3], initial THA procedures are projected to escalate to approximately 640,000 instances between 2018 and 2030. However, the surgical trauma intrinsic to arthroplasty can engender a constellation of adverse postoperative effects. Some studies have delineated acute pain that persists from the sixth to the 24th hour postoperatively [4] and the propagation of an array of inflammatory mediators [2, 5]. These complications further exacerbate postoperative nausea, fatigue, and vomiting, impede swift functional recovery, and can precipitate severe outcomes, such as delirium and, in extreme cases, death. In the preceding two decades, advances in surgical techniques and the integration of rapid recovery principles in THA have yielded remarkable outcomes. However, the employment of glucocorticoids in THA's rapid recovery protocol has sparked controversy. Fewer studies have probed into the use of intermediate-acting glucocorticosteroids like methylprednisolone in THA, with sporadic randomized controlled trials and meta-analyses [6, 7] providing tentative confirmation of its utility in attenuating inflammatory markers, mitigating nausea and vomiting, and assuaging postoperative pain and fatigue during various perioperative stages. However, the question of whether this medication impacts blood glucose fluctuations [8, 9] remains unresolved, necessitating further data-backed exploration of its safety profile. Thus, this study embarks on investigating the therapeutic influence of methylprednisolone, in conjunction with tranexamic acid, as a perioperative intervention in THA. The observational indices include C-reactive protein (CRP) and Interleukin-6 (IL-6) for gauging the anti-inflammatory effects, and the Visual Analogue Scale (VAS) score for assessing the patient's pain intensity. Pain levels and analgesic consumption (specifically, tramadol and meperidine hydrochloride) will be recorded to evaluate the analgesic effect. Moreover, we will monitor postoperative blood glucose fluctuations and quantify the incidence of postoperative nausea, vomiting, and corresponding antiemetic drugs (metoclopramide and ondansetron) administered. Secondary outcomes encompass the International Chronic Fatigue Syndrome (ICFS) score, range of motion (ROM), total blood loss, transfusion rate, length of stay postoperation (post-LOS), postoperative hemoglobin, hematocrit (HCT), coagulation levels, and complications. These will help evaluate the potentially enhanced efficacy of this approach in expediting rehabilitation in THA patients.

## Materials and methods

### Study design

Our study adhered strictly to the protocols established by the Institutional Ethics Review Board of our institution and complied meticulously with the Guidelines on Standards for Reporting of Trials (CONSORT). We enrolled all patients undergoing initial Total Hip Arthroplasty (THA) in this study. However, we established exclusion criteria that included: methylprednisolone or tranexamic acid allergy, rheumatoid arthritis, existing infections, Body Mass Index (BMI) exceeding 30 kg/m2, one-stage bilateral hip replacement, revision surgery, coagulation abnormalities, hematological disorders, malignancies, severe cardio-pulmonary disorders, critical hepatic and renal dysfunctions, history of thrombo-embolism, preoperative hemoglobin less than 90 g/L, and usage of anticoagulation drugs within a week prior to surgery. The participants were randomly allocated to either the Tranexamic Acid (TXA) group or the Methylprednisolone plus Tranexamic Acid (MP + TXA) group as part of a single, single-blind, randomized controlled trial. Patient assignment was randomly ordered based on parity prior to surgery. We obtained written informed consent from each participant.

### Intervention

In the control group (TXA group, *n* = 40), we administered 1 g of tranexamic acid dissolved in 100 ml of saline intraoperatively and then 3 h post-surgery intravenously. For the experimental group (MP + TXA, *n* = 40), we gave an intravenous infusion of 40 mg methylprednisolone in 100 ml saline following the commencement of anesthesia, repeated 24 h later. The control group received only an equivalent dose of normal saline. Both the operating theatre anesthetist and nursing staff were not involved in this study, and the other healthcare personnel assisting in the study was blinded to the treatment allocations.

### Perioperative management

All surgeries were conducted by senior orthopedic surgeons in a class-100 laminar flow operating theatre, employing either general or epidural anesthesia with a posterolateral surgical approach. The hip prostheses implanted were all uncemented and we did not place any drainage catheters.

Postoperatively, the patients were transferred to the post-anesthetic recovery room, and the surgical site was padded routinely upon their return to the inpatient ward. We initiated intermittent orthopedic limb compression therapy until the patients commenced functional out-of-bed exercises. Guided by a rehabilitation therapist, all patients engaged in daily functional and walking exercises. Our strategy for managing pain and PostOperative Nausea and Vomiting (PONV) involved administering preoperative oral diclofenac sodium (100 mg tid) for hyperalgesia. Postoperative pain was evaluated using a 0–10 Visual Analogue Scale (VAS), with intramuscular tramadol (100 mg qd or bid) added when VAS exceeded 4. For severe pain (VAS > 6), intramuscular meperidine hydrochloride (100 mg qd) was given. Metoclopramide (10 mg intramuscularly) was employed as the first-line treatment for severe nausea (VAS > 4) or two or more episodes of PONV. All patients were prescribed subcutaneous naltrexone heparin calcium (0.3 mL, 2850 IU), with subsequent full doses given at 24-h intervals. Post-discharge, we administered rivaroxaban (10 mg orally qd) for 10 days to mitigate the risk of deep vein thrombosis of the lower extremities. Regular Doppler ultrasound and CT scans were used to detect thrombosis in the deep veins of the lower extremities and diagnose pneumonia or pulmonary embolism, respectively.

### Outcome measurements

Our primary outcome parameters included inflammatory marker (CRP and IL-6) levels at 24, 48, and 72 h post-surgery in both groups. We evaluated patients' pain levels using the VAS score, and we recorded both the quantity of pain and the amount of analgesics (tramadol and meperidine hydrochloride) used to assess the analgesic effect. We monitored postoperative blood glucose fluctuations, the incidence of postoperative nausea and vomiting, and the quantity of antiemetic medication (metoclopramide and ondansetron) used. Secondary outcomes encompassed fatigue scale (ICFS) and Range of Motion (ROM), total blood loss (calculated using the Gross formula), transfusion rates, postoperative length of stay (LOS), postoperative haemoglobin, Hematocrit (HCT) and coagulation values, and complications.

### Statistical analysis

For statistical analysis, we employed SPSS version 22.0. We reported continuous variables and mean standard deviations with a 95% confidence interval. We applied the Wilcoxon Mann–Whitney U test for numerical variables not normally distributed or anisotropic. We compared categorical variables using the Pearson χ2 test or Fisher's exact test. We considered p-values less than 0.05 to be statistically significant.

## Results

### Patient selection and baseline information

**Fig. 1:**
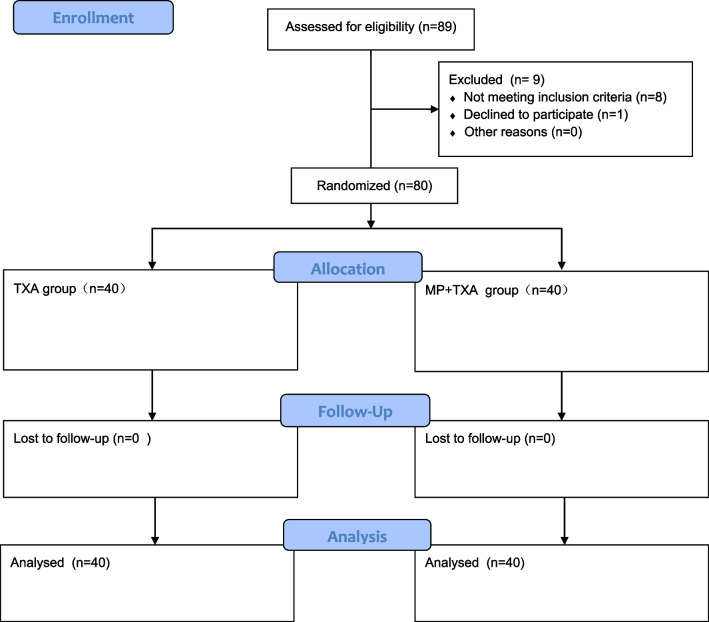
CONSORT flow diagram. MP: Methylprednisolone; TXA: Tranexamic acid

Out of 89 consecutively screened patients, a final cohort of 80 was selected for participation in this study, following a six-month follow-up period and the application of exclusion criteria that removed 8 individuals. A further individual declined to participate. Of the remaining patients, 40 were allocated to the TXA group, while the remaining 40 were assigned to the MP + TXA group. Both primary and secondary outcomes were fully tracked over the follow-up period. Comparative baseline characteristics for both groups are provided in Table 1, and no significant statistical variance was observed (Fig. 1).

**Table 1:** Baseline information

Variables	TXA(40n)	MP + TXA(40n)	*P*
Age(y)	68.45 ± 3.72	68.01 ± 4.63	0.641
Male/Female (n)	19/21	15/25	0.497
Height (cm)	159.56 ± 6.89	160.23 ± 7.16	0.671
Weight (kg)	63.97 ± 4.43	64.23 ± 4.50	0.795
BMI	24.16 ± 2.19	24.56 ± 2.32	0.43
Diabetes (Y/N)	14/26	17/23	0.646
Hypertension (Y/N)	Aug-32	Oct-30	0.789
Diagnosis(DHH/ONFH/OA)	9/16/15	6/11/23	0.201
ASA			
	2.19 ± 0.35	2.11 ± 0.31	0.283
Operation time (min)			
	90.12 ± 4.09	89.56 ± 3.96	0.536
Pre-ICFS			
	62.79 ± 4.52	63.79 ± 5.01	0.351
Pre-VAS scores at rest			
	5.23 ± 0.76	5.46 ± 0.81	0.194
Pre-VAS scores at walking			
	7.56 ± 0.61	7.67 ± 0.73	0.467
Pre-HCT(L/L)			
	40.65 ± 1.98	40.34 ± 1.82	0.468
Pre-ROM			
	92.21 ± 4.13	91.56 ± 3.64	0.457
Pre-HB(g/L)			
	107.93 ± 6.31	106.87 ± 6.06	0.446
Pre-CRP			
	4.34 ± 1.29	4.56 ± 1.36	0.46
Pre-IL-6			
	2.34 ± 1.39	2.37 ± 1.43	0.067
Pre-APTT			
	34.12 ± 3.86	35.74 ± 4.21	0.077
Pre- D-dimer			
	7.68 ± 2.56	7.93 ± 2.71	0.673

MP: Methylprednisolone; TXA: Tranexamic acid; n: number; CRP: C-reactive protein; IL-6: interleukin 6; Hb: Hemoglobin; Hct: Hematocrit; BMI: Body mass index; ICFS: Identity consequence fatigue scale; ASA: America anesthesia association; OA: Osteoarthritis; ONFH: Osteonecrosis of the femoral head; DDH: Development dysplasia hip; ROM: Range of motion; VAS: Visual analog scale; ASA: America anesthesia association; APTT: Activated partial thromboplastin time; y: Years; Pre-: Preoperative

### Inflammatory markers: CRP and IL-6 levels

**Fig. 2:**
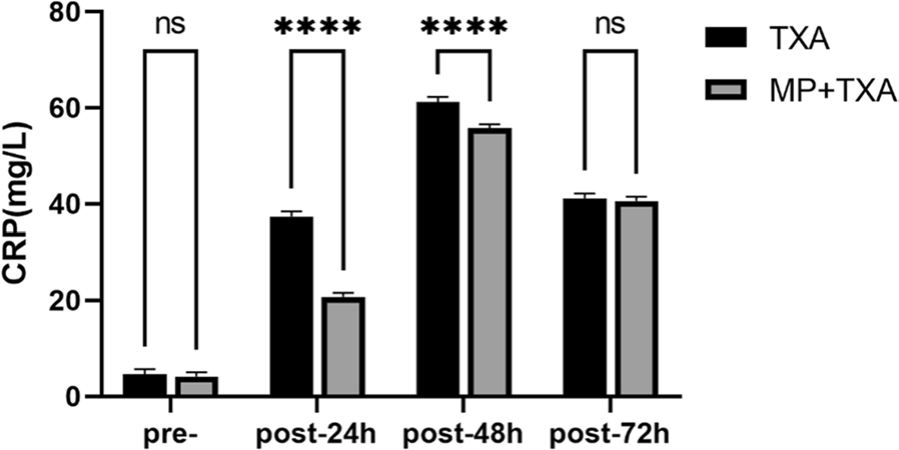
Level of CRP in the two groups. MP:Methylprednisolone; TXA: Tranexamic acid Pre-, preoperative, post, postoperative. ****P < 0.001. ns: not statistically different

**Fig. 3:**
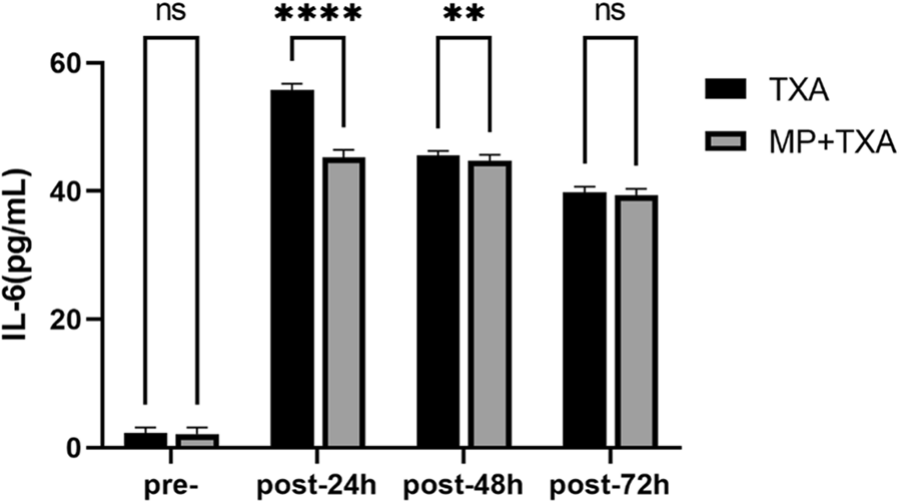
Level of IL-6 in the two groups. MP:Methylprednisolone; TXA: Tranexamic acid Pre-, preoperative, post, postoperative. ****P < 0.001,**P < 0.05. ns:not statistically different

Postoperative levels of CRP and IL-6 showed an increase in comparison with preoperative levels in both treatment cohorts. Peak CRP levels were observed at 48 h postoperative; however, the MP + TXA group demonstrated lower levels at 24 h (*P* < 0.001), 48 h (*P* < 0.001), and 72 h (*P* > 0.05) relative to the TXA group. Similarly, mean IL-6 levels peaked at 24 h postoperatively in both groups, but at 24 h (*P* < 0.001), 48 h (*P* < 0.05) and 72 h (*P* > 0.05), IL-6 levels were reduced in the MP + TXA group compared to the TXA group (as represented in Figs. 2 and 3).

### Pain evaluation and analgesic use

**Fig. 4:**
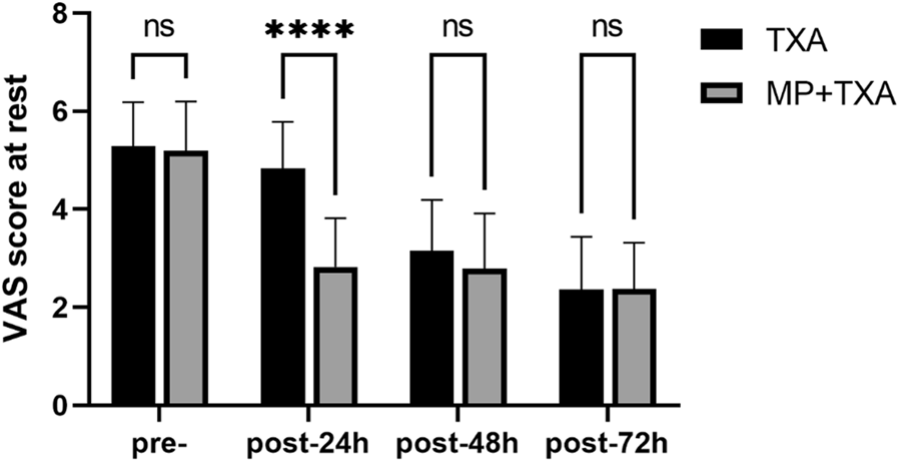
Level of VAS scores at rest in the two groups. MP:Methylprednisolone; TXA: Tranexamic acid Pre-,preoperative; post, postoperative. ****P < 0.001.ns:not statistically different

**Fig. 5:**
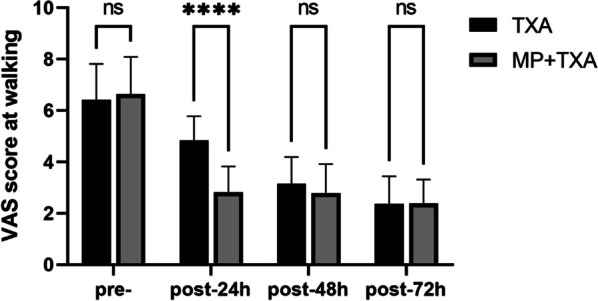
Level of VAS scores at walking in the two groups. MP:Methylprednisolone; TXA: Tranexamic acid Pre-,preoperative; post, postoperative.****P < 0.001;ns:not statistically different

Postoperative VAS pain scores demonstrated a reduction in both treatment groups when compared to preoperative levels. At 24 h postoperatively, the MP + TXA group reported lower pain scores during both rest (*P* < 0.001) and ambulation (*P* < 0.001) relative to the TXA group. However, no statistically significant differences were observed between the two groups for rest and walking VAS scores at 48 and 72 h postoperatively (Figs. 4 and 5). Fewer patients in the MP + TXA group required rescue analgesics, with lower overall consumption of tramadol (*P* = 0.003, and *P* < 0.001, respectively) and meperidine hydrochloride (*P* = 0.025, and *P* < 0.001, respectively) (refer to Table 2).

**Table 2:** Consumption of analgesia and pain relief

Variables	TXA (40个)	MP + TXA (40个)	*P*
Tramadol			
Number of patients (n)	23	10	0.003
Total dose (mg)	3300	1300	< 0.001
Meperidine hydrochloride			
Number of patients (n)	12	4	0.025
Total dose (mg)	1200	400	< 0.001
Metoclopramide			
Number of patients (n)	16	5	0.005
Total dose (mg)	240	80	< 0.001
Ondansetron			
Number of patients (n)	5	2	0.235
Total dose (mg)	25	10	> 0.05

MP: Methylprednisolone; TXA: Tranexamic acid; n: Number

### Postoperative nausea, vomiting, and antiemetic use

The incidence of postoperative nausea and vomiting (PONV) was significantly lower in the MP + TXA group compared to the TXA group (*P* = 0.009) (refer to Table 3). Additionally, fewer patients in the MP + TXA group required the antiemetic metoclopramide, and its overall consumption was also lower (*P* = 0.005, and *P* < 0.001, respectively) (refer to Table 2).

**Table 3:** Secondary outcome indicators and complications

Variables	TXA(40n)	MP + TXA(40n)	*P*
PONV(n)	15	5	0.009
Post-ICFS	70.59 ± 6.12	67.45 ± 5.06	0.015
Post-ROM (°)	96.36 ± 4.87	99.27 ± 5.91	0.018
Post-LOS (day)	5.96 ± 0.93	5.62 ± 0.79	0.082
Post-HB(g/L)	92.53 ± 4.21	93.01 ± 4.25	0.613
Post-HCT(L/L)	45.37 ± 2.63	46.01 ± 2.98	0.312
Post- APTT	30.12 ± 2.79	30.35 ± 2.81	0.714
POST- D-dimer	12.36 ± 5.18	11.68 ± 4.97	0.551
Total blood loss (mL)	963.01 ± 65.68	959.39 ± 61.35	0.799
Blood transfusion rate(n)	6	4	0.49
DVT (n)	0	0	0
PE (n)	0	0	0
Pneumonia(n)	2	0	0.152
Wound infection(n)	0	1	0.314
Gastrointestinal Hemorrhage(n)	0	0	0
Delirium(n)	1	0	0.314

PONV: Postoperative nausea and vomiting; ICFS: Identity-con-sequence-fatigue-scale; ROM: Range of motion; LOS: Length of hospital stay; Hb: Hemoglobin; Hct: Hematocrit; DVT: Deep vein thrombosis; PE: Pulmonary embolism; n: Number; APTT: Activated partial thromboplastin time; post: Postoperative

### Blood glucose variability

**Fig. 6:**
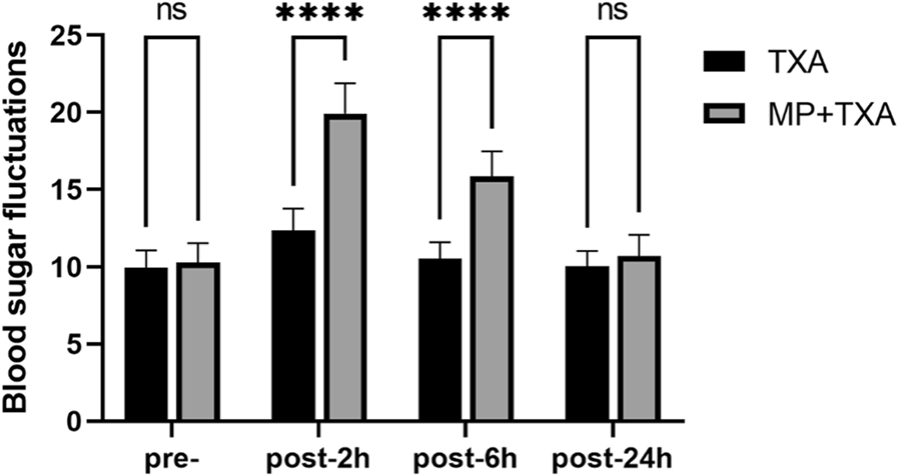
Level of Blood sugar fluctuations in the two groups. MP:Methylprednisolone; TXA: Tranexamic acid; Pre-, preoperative; post, postoperative.****P < 0.001.ns:not statistically different

There was no statistically significant difference in preoperative blood glucose profiles between the two groups (*P* > 0.05). However, postoperative blood glucose variability at 2 and 6 h was significantly higher in the MP + TXA group relative to the TXA group (*P* < 0.001), though these levels normalized by 24 h postoperative (refer to Fig. 6).

### ICFS and ROM evaluation

Preoperatively, a comparison of the ICFS and ROM metrics between the two groups demonstrated no significant variation (*P* > 0.05). However, postoperatively, the ICFS in the MP + TXA group was found to be statistically different from the TXA group (*P* = 0.015) (refer to Table 3). Furthermore, a noteworthy reduction in hip mobility was observed in the MP + TXA group when compared to the TXA group postoperatively (*P* = 0.018) (refer to Table 3).

### Blood loss, transfusion rates, and postoperative hemoglobin and hematocrit levels

There were no significant differences observed between the two groups with regards to total blood loss (*P* = 0.799), transfusion rates (*P* = 0.449), postoperative hemoglobin levels (Post-Hb, *P* = 0.613), and postoperative hematocrit levels (Post-HCT, *P* = 0.312). The length of stay postoperative (Post-LOS, *P* = 0.082) and postoperative coagulation status also did not differ significantly between the groups (refer to Table 3). It is noteworthy that no intraoperative infection or gastrointestinal bleeding complications were observed in either group (refer to Table 3).

## Discussion

In our research, we delved into the possibility of MP and TXA amalgamation to additionally lessen postoperative inflammatory reactions and stimulate swift recuperation. As far as we are aware, there is a lack of extensive research examining the effectiveness and safety of MP and TXA combined usage in patients undergoing total hip replacement procedures. The principal discoveries of our research indicate that the MP and TXA combination resulted in a significant decrease in postoperative CRP and IL-6 measurements, enhanced analgesic response, reduced the frequency of PONV and postoperative fatigue, expedited patients' rapid recovery, and did not heighten the risk of perioperative infections or gastrointestinal bleeds.

Earlier investigations [10–12] have clearly shown a robust connection between perioperative inflammation responses and early postoperative recuperation and potential complications. In our study, the intraoperative and 24 h postoperative administration of 40 mg methylprednisolone notably lessened the postoperative CRP and IL-6 indices. Our findings mirror those of earlier research [13, 14], confirming its efficacy in dampening inflammatory responses and pain, subsequently hastening patient recovery. An extensive study by Guoming Liu [15] et al. carried out a systematic review and meta-analysis regarding the use of MP in advanced osteoarthritis patients undergoing TKA or THA, encompassing six clinical experiments with 350 patients. The outcomes indicated a notable reduction in the inflammatory marker CRP (WMD = − 37.68; 95%CI = − 57.07 ~ − 18.28; *P* = 0.0001) in the MP group at 24 h, which in sequence provided efficient analgesia, lessened the frequency of PONV and postoperative fatigue scores, and speeded up patient recovery. Furthermore, TXA [16], a man-made lysine analogue, which impedes fibrinolysis by competitively blocking the binding site of fibrinolytic pyrrolidine lysine, is commonly utilized in joint replacement surgeries to lessen perioperative blood loss [16, 17]. It is well-established for its hemostatic efficacy, and numerous research studies [18] have substantiated that the critical role fibrinolytic enzymes play in connecting the fibrinolysis and inflammation pathways. It instigates inflammation through the encouragement of cytokine and other inflammatory mediator release from diverse cell types. TXA has also been documented [18, 19] to exhibit an anti-inflammatory response. In our research, contrary to most studies concerning the singular use of TXA or MP, we proposed the hypothesis that the conjunction of TXA and MP would offer synergistic and extra advantages in diminishing the inflammatory response post-primary unilateral hip arthroplasty as opposed to the sole use of TXA. This hypothesis was affirmed by our findings, which demonstrated that the collaborative treatment approach attained lower CRP and IL-6 readings. Prior research has highlighted the efficacy of glucocorticoids in mitigating postoperative pain following THA. Lunn et al. [20] evidenced that patients subjected to MP treatment experienced more substantial pain relief following THA than those in control groups. Consequently, the combined intervention of MP + TXA might offer more effective pain relief for patients following THA as compared to exclusive TXA treatment. However, prior investigations have highlighted that TXA possesses anti-inflammatory attributes and confers pain relief. Importantly, some patients continue to report pain symptoms in the postoperative period. As indicated in prior reports [21], the pain-relieving impact of glucocorticoids is accomplished by inhibiting phospholipases, hence blocking the cyclooxygenase and lipoxygenase pathways in the inflammation cascade. Additionally, it suppresses tissue kinin levels and diminishes the liberation of neuropeptides from nerve endings, resulting in the reduction in tissue inflammation and pain. Consequently, the perioperative usage of MP may potentially compensate for the lack of anti-inflammatory efficacy of TXA, further alleviate pain, and hasten patient recuperation. It additionally results in reduced utilization of tramadol and meperidine hydrochloride post-THA.

Several factors contribute to postoperative nausea and vomiting (PONV), including surgical procedures, individual predisposition, the use of anesthesia and analgesic drugs, and importantly, the stimulation of inflammation. Recent studies suggest that the congregation of immune cells and inflammatory mediators, triggered by surgical trauma, may incite postoperative intestinal paresthesia or even lead to full-blown intestinal obstruction [22]. Hence, it is postulated that targeting this inflammatory cascade might reduce PONV occurrence. Existing research lends credibility to this theory, citing the demonstrated antiemetic effects of curbing this inflammation, predominantly achieved by regulating prostaglandin synthesis or inhibiting the release of endogenous opioid-like substances [23]. Our research substantiated these theories; observing significantly less PONV incidents in the TXA + MP group compared to the TXA group alone (*P* < 0.05). This finding suggests that the administration of an additional dose of MP 24 h post-surgery could potentially diminish the incidence of PONV. Regarding postoperative fatigue, research conducted by Katarina et al. [24] demonstrated that postoperative physiological stress led to an upsurge in inflammatory markers, directly stimulating the nucleus tractus solitarius and the vagus nervous system, thus exacerbating postoperative fatigue. Consequently, minimizing the central nervous system's exposure to these inflammatory factors is of paramount importance. In line with this, our research noted a statistically significant decrease in postoperative fatigue in the TXA + MP group compared to the control group (*P* = 0.015). Further studies affirm this observation, indicating a significant role of inflammatory markers such as IL-6 and TNF-α in the decline of muscle strength and the onset of myasthenia gravis [25]. In fact, a study by Amanda et al. [26] discovered that elderly inpatients with concurrent inflammation and elevated IL-6 levels at the time of acute infection exhibited a drastic decrease in muscle strength and endurance compared to their non-inflammatory counterparts. Also, in terms of postoperative range of motion (ROM), we found a statistically significant improvement in the experimental group compared to the control group (*P* = 0.018).

However, the appropriate dose of MP in patients undergoing total hip arthroplasty (THA) remains contentious [27]. Some studies propose an initial dose range of 40 to 125 mg for THA, yet it has been observed that patients receiving lower doses of MP may still struggle with pain, fatigue, and PONV. Our study is underpinned by the hypothesis that low-dose MP may fall short of meeting anti-inflammatory needs given its half-life of approximately 24 h. Thus, administering an additional 40 mg dose of MP 24 h post-surgery seems plausible. When used in tandem with TXA, this regimen has shown promise in attenuating postoperative inflammation and pain without compromising the hemostatic efficacy of TXA.

Glucocorticoids administered preoperatively may induce hyperglycemia during the perioperative period [28]. Studies have suggested that local glucocorticoid application might heighten the risk of postoperative infections. Even though the infection rate did not exhibit a statistically significant difference compared to the control group, this potential complication warrants consideration [29]. Gądek et al. [7] reported a statistically significant surge in blood glucose levels in the experimental group versus the control group following perioperative use of MP in joint replacement procedures. This was noticeable at both 2 h and 6 h postoperatively, which aligns with our findings. Our study also revealed that while blood glucose levels spiked at 2 and 6 h postoperatively (*P* < 0.001), they normalized by the 24 h mark. Conversely, research by Lindberg-Larsen et al. [30] concluded that MP minimally affected blood glucose variability and that normal levels could be re-established within a day.

While the beneficial clinical efficacy of MP has been supported by numerous prior studies [7, 31, 32], the potential for elevated adverse effect risks remains a topic of debate. In our study, methylprednisolone was administered both intraoperatively and postoperatively, with no observed surgical site infections, gastrointestinal bleeding, or thrombosis during the monitoring period. Still, larger-scale prospective studies are warranted to thoroughly assess the safety of the combined TXA + MP regimen. This study has certain limitations: namely, the short follow-up period precluded a comprehensive safety evaluation of TXA + MP, requiring future studies with longer follow-ups. Although, the safety of intravesical TXA and MP usage in THA patients has been previously substantiated. Also, our sample size was relatively small (40 patients per group), potentially weakening the study's robustness. Lastly, the optimal dosing and duration of the combined TXA + MP regimen remain undetermined and warrant further investigation.

## Conclusion

In this study, we showed that MP combined with TXA can significantly reduce postoperative CRP and IL-6 levels, alleviate postoperative pain, promote analgesia, reduce the incidence of POVN; the patient's postoperative experience is good, and does not affect to aggravate the fluctuation of blood glucose, reduce postoperative fatigue, improve hip mobility, and does not increase the incidence of perioperative serious complications of THA.

## Data Availability

Data for this article were sourced from patients hospitalized at our institution for total hip arthroplasty. The data are both credible and reliable.
